# Child Life under Queen Victoria

**Published:** 1897-03-20

**Authors:** 


### Child Life under Queen Victoria.

### VI.—AGRICULTURAL GANGS.

In the early days of the factory agitation, it was a common reproach to cast at those who were demanding shorter hours and better conditions for the factory workers that the state of things was worse in the agricultural counties than in the manufacturing districts. There was some truth in the accusation. The agricultural labourers led hard, laborious, ill-requited lives half a century ago, and even now their condition has not improved in proportion to that of other workers. Many developments of commerce, which have profited almost all the other members of the industrial community, have done nothing for him. Rural labourers are poor, and even a shilling or two which a child's earnings may add to the weekly income is a matter of importance to them. Therefore, they are even less inclined than the average factory worker to accept legislative restrictions to children's work.

It is indeed impossible to lay down hard and fast rules with regard to time and means in agricultural work. Day in, day out, the steam engine can go, and the factory work proceeds with absolute regularity. But in agriculture there are seasons of almost complete stagnation, weeks when the farmer and his men must wait for the frost to break or the snow to melt; and, on the other hand, seasons of over-pressure, when no limit can be fixed to the length of the working day, and every hand must be pressed into the service if the "kindly fruits of the earth" are by any means to be gathered in due season. On the farm it is not possible always to restrict labour within an eight hours' day, and the extra pressure at one time is compensated by extra slackness at another. Neither is it practicable altogether to forbid children's labour. Indeed, there has never been any attempt to keep children from working on their father's gardens or allotments. There is no great harm in their helping occasionally on farms where their fathers are employed. The work is not in itself unhealthy; it is not, as a rule, hard, and the School Board will take care that no child neglects the duty of education for too long a time.

But the case is different when the factory system, with all its faults, save indeed that of ill-ventilated workshops, is introduced into agricultural life. This is what happened with the agricultural gang. Under the system which prevailed until 1867, a gang-master, who was a mere contractor, would hire all the children of a whole neighbourhood, and let out their labour to the highest bidder. Thus the children's work often lay far from their homes, and this in itself made their hours of labour long. They were marshalled in the early morning and sent off to the fields they were to work in. And early morning means early in the country. The present writer knows of girls engaged in gathering fruit who left their homes at four in the morning and did not get back till ten o'clock at night. They took their dinner with them, such as it was, and were allowed little enough time to eat it. But this was the inevitable over-pressure of work that must be done at once if it is to be done at all; it was fairly well paid, and, coming in holiday-time, did not interfere with education.

But the poor little slaves of the gang—the very word gang has a brutal sound—were employed at all seasons of the year. They did weeding, they spread manure, they thinned turnips, they picked stones off the fields. The greater part of their work involved constant stooping; their hands were chapped and swollen with exposure to weather—the turnip thinning blistered them, the stones cut them. These children, some of them not more than six or seven, were old while yet they were young, and suffered those aches and chills which are supposed to be the prerogative of age. It was proved, moreover, that the moral condition of the workers in these gangs was as low as the intellectual; they became degraded and brutalised.

To improve this state of matters, it was enacted in 1867 that no child under eight years of age should be employed in an agricultural gang. A gang was only to be formed by a licensed gang-master, whom two or more justices were satisfied was a person of good character, and fit to be entrusted with the management of such a body of workers. Anyone who, without this licence, collected a gang, was liable to a penalty of twenty shillings for every day that they worked. To prevent any drunken parent from signing away his child's liberty for means of self-indulgence, it is enacted that "no licence shall be granted to any person who is licensed to sell beer, spirits, or any other excisable liquor." In short, the publican is not regarded as fit to be a gang-master. In giving a licence, the Justices are to annex to it a condition limiting in such manner as they think expedient the distances within which the children employed are to be allowed to travel on foot to their work, and any gang-master who makes the children go further afoot than his licence permits is liable to a fine of ten shillings. A very important provision of the Act is that which insists that where girls are employed in the gang they shall be under the charge of a duly-licensed gang-mistress. Women are said to be hard taskmasters to their own sex; but circumstances may arise in which the harshest woman is a better friend than any man. It is also forbidden to employ both males and females in the same gang.

Some critics* seem to think that the Agricultural Gangs Act has accomplished little. It is, as we have said, impossible to bring agricultural labour within such hard and fast lines as factory work, but the provisions of this Act are at least such as to save rural children from any great or permanent wrong.

* Mr. Whately Cooke Taylor in his "Modern Factory System," speaks disparagingly of this Act, which he seems to think has been almost ineffective.

